# PARP-1 Inhibitor, DPQ, Attenuates LPS-Induced Acute Lung Injury through Inhibiting NF-κB-Mediated Inflammatory Response

**DOI:** 10.1371/journal.pone.0079757

**Published:** 2013-11-21

**Authors:** Gang Wang, Xiaojia Huang, Yongjin Li, Kangkang Guo, Pengbo Ning, Yanming Zhang

**Affiliations:** 1 College of Veterinary Medicine, Northwest Agriculture & Forestry University, Yangling, Shaanxi, China; 2 Department of Pharmacology, School of Medical Sciences and Laboratory Medicine, Jiangsu University, Zhenjiang, Jiangsu, China; Universidade de São Paulo, Brazil

## Abstract

Acute lung injury (ALI) is characterized by overwhelming lung inflammation and anti-inflammation treatment is proposed to be a therapeutic strategy for ALI. Poly (ADP-ribose) polymerase-1 has been demonstrated to be involved in tissue inflammation and one of its inhibitors, 3, 4-Dihydro-5[4-(1-piperindinyl)butoxy]-1(2H)-isoquinoline (DPQ), exerts anti-inflammatory effect. However, it is still unclear whether the DPQ possesses the protective effect on ALI and what mechanisms are involved. In this study, we tested the effect of DPQ on the lung inflammation induced by lipopolysaccharide (LPS) challenge in mice. We found that 6 h-LPS challenge induced significant lung inflammation and vascular leakage in mice. Treatment with DPQ at the dose of 10 μg/kg markedly reduced the neutrophil infiltration, myeloperoxidase activity and up-regulation of pro-inflammatory mediators and cytokines. LPS-elevated vascular permeability was decreased by DPQ treatment, accompanied by the inhibition of apoptotic cell death in mice lungs. In addition, we isolated mice peritoneal macrophages and showed pretreatment with DPQ at 10 μM inhibited the production of cytokines in the macrophages following LPS stimulation. DPQ treatment also inhibited the phosphorylation and degradation of IκB-α, subsequently blocked the activation of nuclear factor (NF)-κB induced by LPS *in vivo* and *in vitro*. Taken together, our results show that DPQ treatment inhibits NF-κB signaling in macrophages and protects mice against ALI induced by LPS, suggesting inhibition of Poly (ADP-ribose) polymerase-1 may be a potential and effective approach to resolve inflammation for the treatment of ALI.

## Introduction

Acute lung injury (ALI) is a severe respiratory disorder characterized by strong lung inflammation and increased microvascular permeability [1]. The most common cause of ALI is sepsis resulting from bacterial infection [2]. Current therapeutic strategy includes protective ventilation and supportive fluid conservative [3]. Despite extensive studies about the pathogenesis of ALI have been conducted, the mortality of ALI remains very high [4], [5]. Thus, it is critical to explore the innovative therapies and effective medications for ALI.

Among the animal models established for the investigation of mechanism involved in ALI, intraperitoneal injection of lipopolysaccharide (LPS) is one of the most commonly used models of ALI [6], [7]. LPS is the major component of outer membrane of Gram-negative bacteria, and contributes to the integrity of bacterial structure [8]. LPS can act as endotoxin and bind to its receptor on cell membrane, initiating a series of innate immune responses [9]. In animals, LPS challenge induces rapid infiltration of polymorphonuclear leukocyte, leading to the release of inflammatory mediators, cytokines and chemotactic factors [10], [11]. Subsequently, inflammation is induced in alveolar space and epithelial-endothelial barrier is disrupted, resulting in lung injury. Thus, inhibition of lung inflammation may be an approach for the treatment of ALI.

Recent studies have shown Poly (ADP-ribose) polymerase-1 (PARP-1) plays an important role in regulation and maintenance of tissue inflammation [12]. PARP-1 is the most abundant member of PARP family consisting of 17 enzymes which share a highly conserved PARP signature motif in the catalytic domain [13], [14]. PARP-1 is the most extensively investigated isoform and composed of DNA-binding domain, automodification domain and catalytic domain [15]. Upon recognizing and binding to DNA strand breaks, PARP-1 is activated and functions as the center of stress responses, leading to DNA repair or cell death [16]. In PARP-1 knockout mice, tissue or cellular inflammation are reduced under various pathological conditions, such as pneumococcal-induced meningitis [17], trinitrobenzene sulfonic acid-induced colitis [18], cisplatin-induced kidney inflammation [19]. Accordingly, treatment of PARP-1 by its inhibitors or siRNA inhibits inflammatory responses in experimental allergic encephalomyelitis [20], temporoman-dibular joint disorder [21], and pleuritis [22]. More importantly, chronic obstructive pulmonary disease (COPD) patients exhibit higher PARP-1 activation in lymphocytes compared with healthy individuals, accompanied with higher levels of cytokines in plasma [23], suggesting the positive correlation of PARP-1 activity and chronic inflammation. Although inhibition of PARP-1 may have the potential value for the treatment of tissue injury following inflammatory stimuli, the effects of PARP-1 inhibitor on ALI remain unclear. Herein, we report that a potent and selective PARP-1 inhibitor, DPQ (3,4-dihydro-5-[4-(1-piperidinyl)butoxyl]-1(2H)-isoquinolinone), inhibits macrophage-mediated inflammation and attenuates ALI induced by LPS challenge in mice. Therefore, inhibition of PARP-1 by DPQ may be an efficient approach to inhibit inflammation for the treatment of ALI.

## Materials and Methods

### Antibodies, Reagents and Mice

Rabbit anti-NF-κB-p65 (phospho-p65), rabbit anti-IκB-α, mouse monoclonal anti-GAPDH antibodies were bought from Cell Signaling Technology. PARP-1 inhibitor DPQ was purchased from Santa Cruz Biotechnology. LPS from *Escherichia coli* 0111:B4 was obtained from Sigma-Aldrich.

Eight to ten week-old male C57BL/6 mice were purchased from the Experimental Animal Center of The Fourth Military Medical University (Xi’[an, China) and maintained in a pathogen-free and light-controlled room (12 hours light and 12 hours dark) with free access to food and water. All animal experiments were performed in accordance with protocols approved by the committee for the Ethics on Animal Care and Experiments at Northwest Agriculture & Forestry University.

### Sepsis Model

To induce the mouse model of sepsis, LPS at a dose of 7.5 mg/kg body weight were administered by intraperitoneal (i.p.) injection. Thirty minutes later after LPS administration, DPQ at the doses of 1 or 10 µg/kg body weight was injected into peritoneal cavity of the mice. Vehicle (0.01% dimethyl sulfoxide (DMSO) in PBS)-treated mice served as the control. After 6 hours, mice were anaesthetized with i.p. injection of the mixture of ketamine (100 mg/kg) and xylazine (10 mg/kg), and then sacrificed for subsequent experiments.

### Histological Analysis

Mice were anaesthetized with i.p. injection of the mixture of ketamine (100 mg/kg) and xylazine (10 mg/kg). Following exposure, the lung tissues were perfused by PBS and fixed by gently injection of 10% formalin through trachea. After tracheal ligation, the lung tissues were incubated in 10% formalin overnight. Then, the lung tissues were embedded with paraffin and sectioned at 5 μm thickness and then stained with Hematoxylin & Eosin (H & E staining).

### Myeloperoxidase (MPO) Activity Assay

For MPO activity assay, lung tissues were flushed free of blood by PBS and homogenized in 50 mM phosphate buffer (PB). The homogenates were centrifuged at 14,000 rpm at 4°C for 30 minutes. After discarding the supernatants, the pellets were resuspended in PB buffer containing 0.5% hexadecyl trimethylammonium bromide by vigorously vibrating to break up the large pellets. Then the pellets were frozen at −70°C for 30 minutes and thawed at 37°C. Subsequently the pellets were homogenized and centrifuged again. Thereafter the supernatants were used for MPO activity assay with a kinetic reading at 460 nm for 5 minutes. The results were presented as V-Max value/g lung tissue.

### Vascular Permeability Assessment

To estimate the vascular permeability, 20 mg/kg of Evans blue-conjugated albumin (EBA) was intravenously injected into mice 30 minutes before sacrificing the mice and tissue collection. Lung tissues were perfused with PBS to flush away residual blood, blotted dry and weighted. Subsequently, the lung tissue was homogenized in 1 ml of PBS. The homogenate was then incubated with 2 volumes of formamide at 60°C overnight and centrifuged at 12,000 rpm for 20 minutes. The supernatant was determined with spectrophotometer at 620 nm. With the standard curve of Evens blue dye, the extravasated EBA in lung homogenate was calculated and presented as μg of Evans Blue dye per g of lung tissue.

### Cell Apoptosis Assay

To evaluate apoptic cell death in the mice lungs, lung tissue cryosections (5 μm) were stained with In Situ Cell Death Detection Kit (Roche Diagnostics) according to the manufacturer’s instructions. The nuclei were counterstained with DAPI. Images were obtained using a Zeiss ApoTome microscope. The apoptotic cells were determined by condensed nuclei with bright staining. At least 3 consecutive cryosections were counted for each mouse, and values were expressed as the positive nuclei per 1,000 nuclei.

### Isolation of Abdominal Macrophages and Treatment

Mice at 8–10 weeks old of age were sacrificed by neck break after CO_2_ euthanasia. To collect mouse macrophages, 5–10 ml of pre-cold PBS was injected into peritoneal cavity and flushed twice. The cell suspension was centrifuged and washed once with ice-cold PBS at 4°C. The pellets were resuspended in RPMI 1640 containing 10% fetal bovine serum and seeded onto six-well plates. After overnight incubation, the cells were washed twice with RPMI 1640 and treated with same volume of DMSO or 1, 10 μM DPQ at 37°C for 30 minutes. Then the cells were stimulated with 100 ng/ml LPS at 37°C for indicated time and lysed for further experiments.

### Quantitative Realtime PCR Assay

For RNA extraction from mice lungs, tissues were homogenized in 1 ml of Trizol and total RNA was isolated with an RNeasy Mini kit (Qiagen, Valencia, CA). For macrophages, cells pretreated with DPQ or DMSO were stimulated with 100 ng/ml LPS for indicated time and lysed with buffer from RNeasy Mini kit. Then 1 μg of total RNA was reverse-transcripted into cDNA with a High Capacity Reverse-Transcription kit (Applied biosystems, Foster city, CA). RT-PCR detection was performed with SYBR Green supermix kit (Bio-Rad, Hercules, CA) in a sequence detection system (ABI Prism 7000; Life Technologies, Grand Island, NY). The following primer pairs were used in the analysis: mouse GAPDH, 5′-TGCGACTTCAACAGCAACTC-3′ and 5′-CTTGCTCAG TGTCCTTGCTG-3′; mouse interleukin (IL)-1β, 5′-CGACAAAATACCTGTGGCCT-3′ and 5′-TTCTTTGGGTATTGCTTGGG-3′; mouse IL-6, 5′-GTGTAATTAAGCCTCCG ACTTG-3′ and 5′-CCAGTTGCCTTCTTGGGAC-3′; mouse tumor necrosis factor-α (TNF-α), 5′-TTCTGTCTACTGAACTTCGGGGTGATCGGTCC-3′ and 5′-GTATGAG ATAGCAAATCGGCTGACGGTGTGGG-3′; mouse chemokine (C-X-C motif) ligand-1 (CXCL-1), 5′-CTGGGATTCACCTCAAGAACATC-3′ and 5′-CAGGGTCAAGGCAA GCCTC-3′; mouse macrophage inflammatory protein-2 (MIP-2), 5′-CCAACCACCAGG CTACAGG-3′ and 5′-GCGTCACACTCAAGCTCTG-3′; and mouse inducible nitric oxide synthase (iNOS), 5′-GTTCTCAGCCCAACAATACAAGA-3′ and 5′-GTGGACG GGTCGATGTCAC-3′. The expression of mouse gene was normalized by GAPDH.

### Western Blot Assay

Lung tissues were homogenized in RIPA buffer containing proteinase and phosphatase inhibitors (Sigma-Aldrich, St. Louis, MO). For macrophages, cells were directly lysed by RIPA buffer and sonicated for 10 seconds. The lysate was centrifuged at 15,000 rpm for 30 minutes at 4°C and the supernatant was collected. Then the protein concentration was determined with BCA protein assay kit (Thermo Fisher Scientific, Rockford, IL). Each sample containing 30 μg of total protein was loaded and separated by 12% SDS-PAGE gel. After the protein was electrotransferred onto nitrocellulose membranes, the membranes were blocked with 5% skim milk and incubated with antibodies overnight at 4°C. Membranes were then incubated with horseradish peroxidase-conjugated secondary antibodies. The immunoblots were visualized with an enhanced chemiluminescence reagent (Thermo Fisher Scientific, Waltham, MA). The intensity of each band was quantified with NIH Image J software (Bethesda, MD).

### Statistical Analysis

Data were expressed as mean ± SEM. Differences between groups were examined for statistical significance using Student’s *t*-test or one-way *ANOVA* with Bonferroni correction. A *P* value less than 0.05 was considered statistically significant.

## Results

### DPQ Treatment Ameliorated Severe Lung Inflammation Induced by LPS

**Figure 1:**
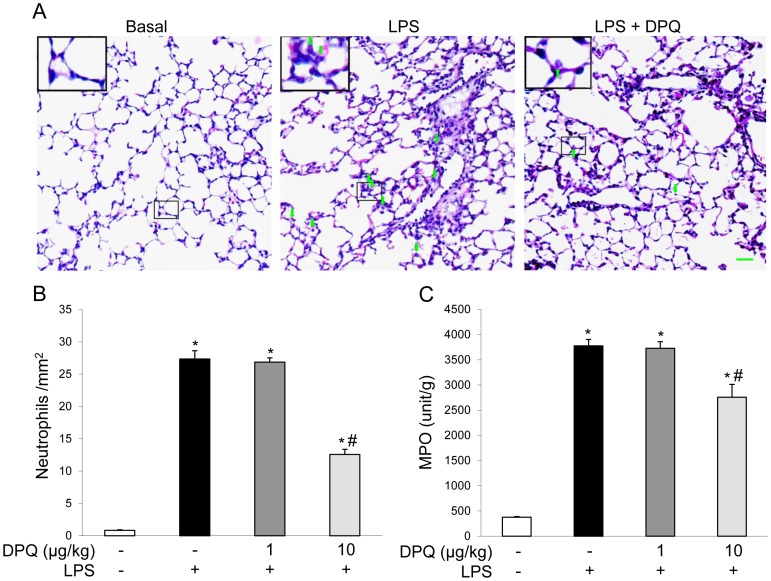
DPQ treatment attenuates LPS-induced acute lung injury. To induce the ALI, 8–10 weeks old male C57BL/6 mice were intraperitoneally injected with LPS at 7.5 mg/kg. After 30 minutes, 1 or 10 μg/kg of DPQ was administrated. (A) Representative micrographs of H & E staining (DPQ 10 μg/kg). Lung tissues were fixed by formalin and embedded into paraffin. Following sectioning with 5 μm thickness, the sections were stained with H & E. Green arrows indicate the infiltrated neutrophils. Scale bar: 50 μm. (B) Quantitative analysis of recruited neutrophils in lungs at 6 hours after LPS challenge. The average number of infiltrated neutrophils are shown. (C) MPO activity assay of lung tissue. Lung tissues were homogenized, and the MPO activity of homogenates were measured with microplate reader at 460 nm. Data are expressed as mean ± SEM; n = 5; *P<0.05 versus Basal; #*P*<0.05 versus LPS alone.

LPS-induced ALI animal model is a commonly used experimental model for investigating molecular mechanisms in ALI [24]. To determine the effect of DPQ treatment on ALI, mice were first challenged with LPS for 6 hours and injected with 1 or 10 μg/kg of DPQ 30 minutes after LPS administration. H&E staining of the lung sections showed increased thickness of alveolar walls and neutrophil infiltration in mouse lungs after LPS-challenge. Compared to PBS-treated lungs, DPQ at 10 μg/kg treated lungs exhibited less neutrophil sequestration and structural damage (**Fig. 1A and 1B**), while DPQ treatment at 1 μg/kg showed no effect. Next, we assessed the lung inflammation by examining the MPO activity, an indicator of neutrophil infiltration [11]. We found 6 h-LPS challenge resulted in high MPO activity in PBS-treated mouse lungs. DPQ at 10 μg/kg significantly decreased the lung MPO activity following LPS challenge (**Fig. 1C**).

### DPQ Inhibited the Up-regulations of Pro-inflammatory Mediators and Cytokines in Mice Lungs Following LPS Challenge

**Figure 2:**
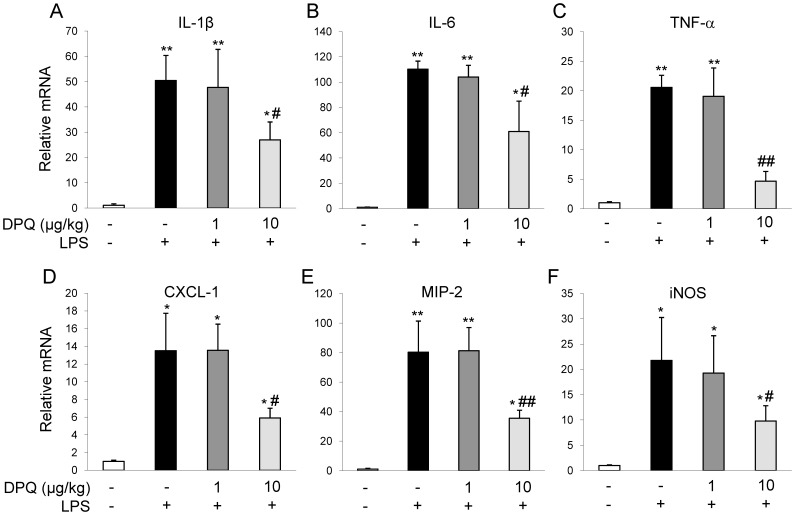
The mRNA expression of inflammatory mediators and cytokines in lung tissues. Lung tissues were collected and homogenized for RNA isolation and QRT-PCR analysis was performed to examine the mRNA expressions. Data presents as mean ± SEM; n = 5; *P<0.05 and **P<0.01 versus Basal; #*P<*0.05 and ##*P<*0.01 versus LPS alone.

After LPS challenge, pro-inflammatory mediators in mouse lungs will be elevated, which include TNF-α, interleukins, MIP-2 and CXCL-1 [1]. Thereafter neutrophils, monocytes and lymphocytes will be recruited into alveolar space and cause severe lung inflammation. To determine the expressions of these mediators, we isolated mRNA from the lung tissues and performed QRT-PCR assay. We found the mRNA expressions of TNF-α, IL-1β, IL-6, MIP-2, iNOS and CXCL-1 were markedly increased in mouse lungs 6 hours post-LPS challenge, which were inhibited by DPQ treatment at 10 μg/kg (**Fig. 2**).

### DPQ Restored the Lung Vascular Permeability and Cell Apoptosis

**Figure 3:**
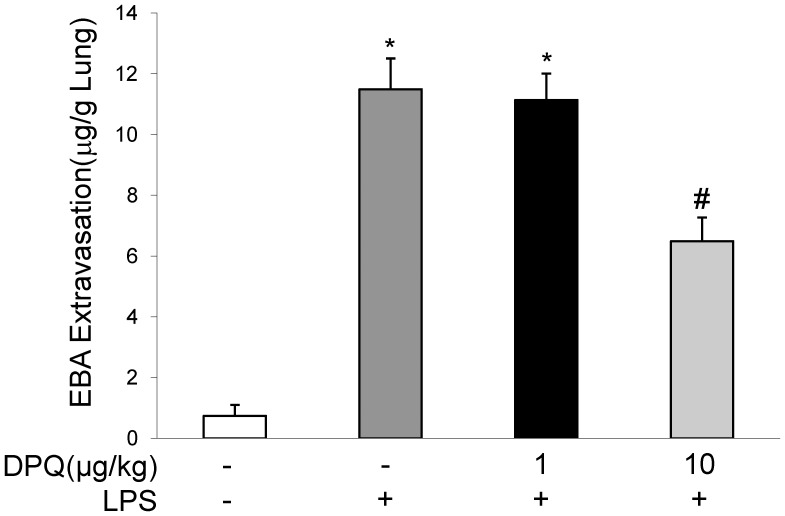
Analysis of lung vascular permeability after LPS challenge. EBA was intravenously injected 30 minutes before tissue collection. The extravasated EBA was measured and presented as µg of EBA per g lung tissue. Data are expressed as mean ± SEM; n = 5; *P<0.01 versus Basal; #*P<*0.01 versus LPS alone.

During ALI induced by LPS, lung endothelial cells undergo apoptosis and vascular permeability increases, peaking at 6–12 hours post-LPS challenge [10], [25]. To confirm the effects of DPQ on lung injury, we determined the lung vascular permeability by EBA assay. EBA was intravenously injected into the mice and EBA extravasation in lung tissue was measured. EBA influx was significantly increased 6 hours post-LPS challenge, as compared with control group. DPQ treatment at 10 μg/kg decreased the EBA extravasation (**Fig. 3**).

**Figure 4:**
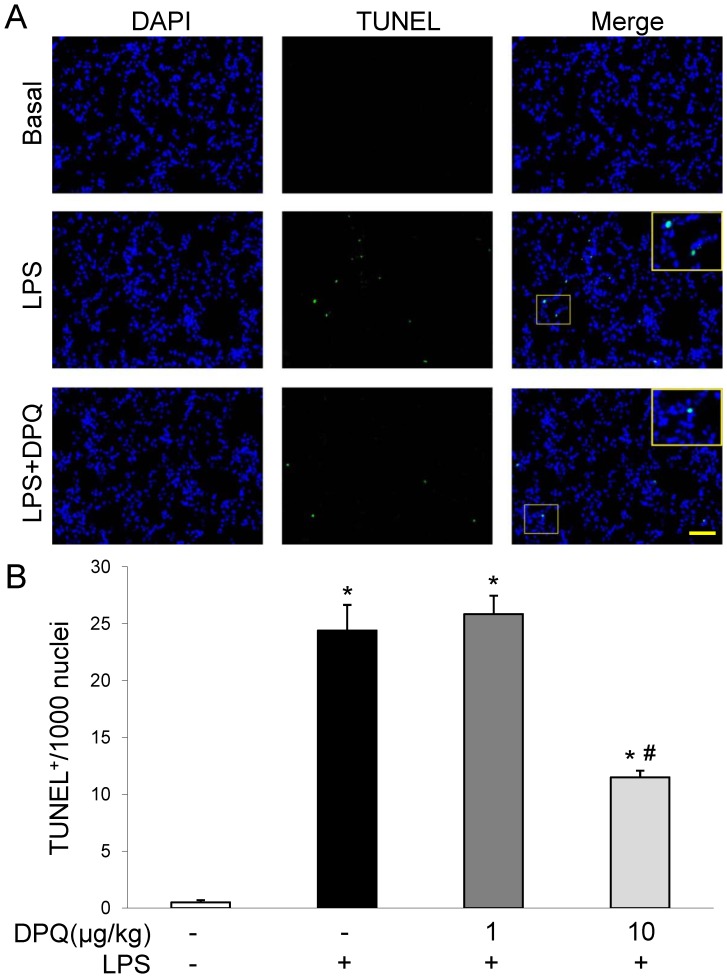
Cell apoptosis in mice lungs following LPS challenge. Cryosections of lungs collected at 6 hours after LPS challenge were stained with FITC-conjugated TUNEL to identify apoptotic cells; nuclei were counterstained with DAPI. (A) Representative micrographs of TUNEL staining (DPQ 10 μg/kg). Scale bar: 50 μm. (B) Quantitative analysis of apoptotic cells in mouse lungs at 6 hours after LPS challenge. The number of TUNEL-positive nuclei from 3 consecutive cryosections from each mouse were averaged. Data are expressed as mean ± SEM; n = 5; *P<0.01 versus Basal; #*P<*0. 01 versus LPS alone.

Next, we examined the apoptotic cell death in lung tissues by Terminal deoxynucleotidyl transferase-mediated dUTP nick end labeling (TUNEL) staining. The apoptotic cells show condensed and fragmented nuclei with TUNEL staining. We found the mice treated with LPS showed markedly increased apoptotic cells in lung tissues, while DPQ at 10 μg/kg inhibited the cell apoptosis induced by LPS (**Fig. 4**).

### DPQ Inhibited Degradation of IκB-α and Activation of NF-κB in Mice Following LPS Challenge

**Figure 5:**
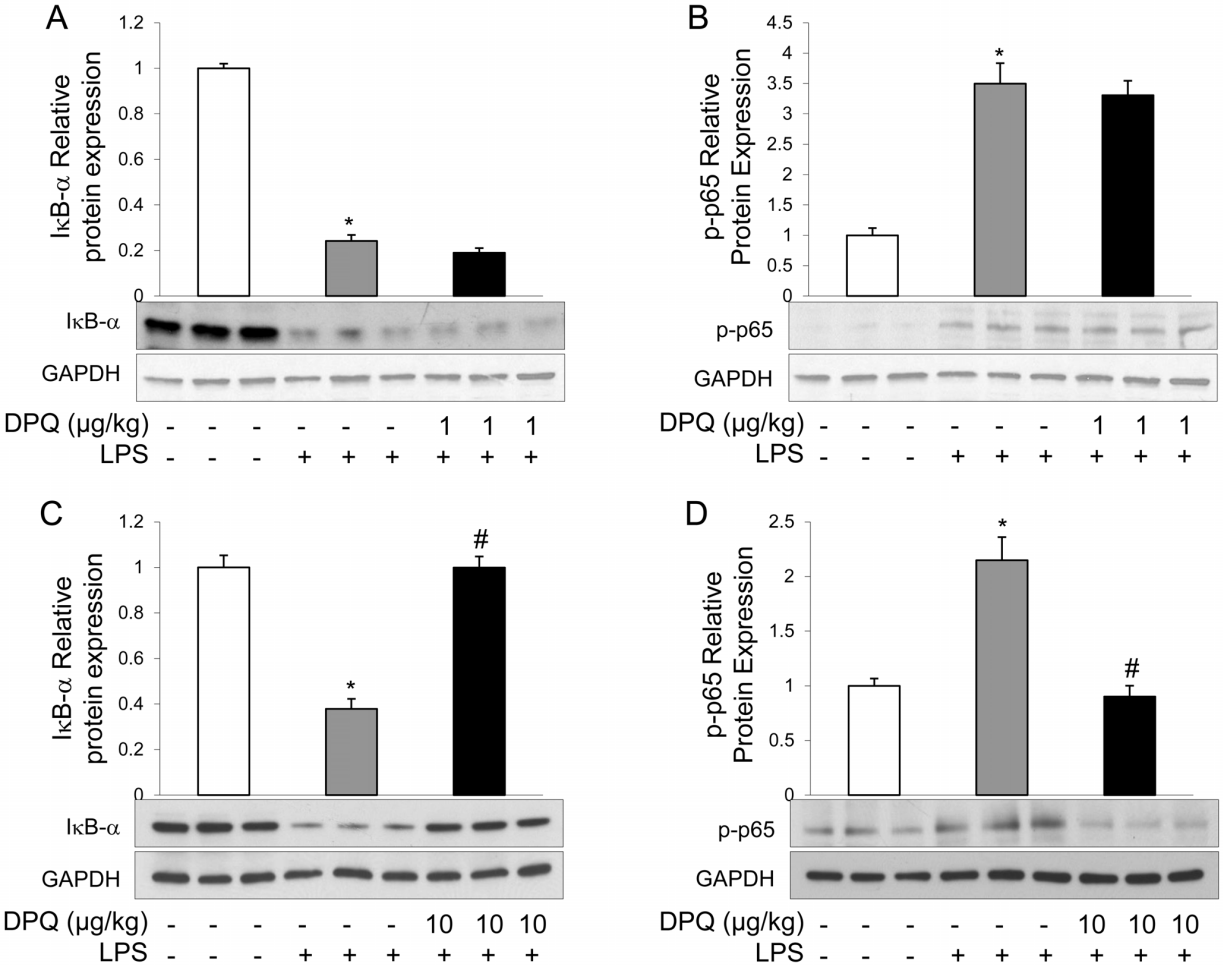
DPQ treatment blocks degradation of IκB-α and subsequent activation of NF-κB. After the mice were treated with LPS for 6 hours, the lung tissues were collected and homogenized for the extraction of total proteins. The expressions of IκB-α (A, C) and phosphorylation of NF-κB p65 (B, D) were detected by Western blot. The experiment was repeated for 3 times with similar results. Lower panel shows the representative immunoblot and upper panel shows the quantitative analysis of the protein expression. Data are expressed as mean ± SEM; n = 3; *P<0.01 versus Basal; #*P*<0.01 versus LPS alone.

Nuclear factor (NF)-κB signaling plays a central role in the initiation and regulation of cellular inflammatory response to bacterial stimuli. In resting condition, IκB-α binds to NF-κB complex and prevents its activation; following phosphorylation and degradation of IκB-α, NF-κB is released, phosphorylated and translocated into nucleus, resulting in the increased expressions of pro-inflammatory mediators [26]. Thus, to investigate the mechanisms in which DPQ enhanced lung inflammation resolution, we assessed the effects of DPQ at 1 or 10 μg/kg on the degradation of IκB-α and activation of NF-κB by western blot analysis. The mice treated with LPS exhibited significant degradation of IκB-α in lungs, whereas DPQ treatment at the dose of 10 μg/kg rather than of 1 μg/kg prevented the IκB-α degradation (**Fig. 5A, 5C**). In contrast, LPS challenge induced the phosphorylation of NF-κB p65 subunit, compared with basal group. DPQ treatment at 10 μg/kg inhibited the up-regulation of phosphorylated NF-κB p65 (**Fig. 5D**), whereas 1 μg/kg of DPQ-treatment had no effect on the activation of p65(**Fig. 5B**).

### DPQ Pretreatment Reduced the Production of Pro-inflammatory Mediators and Inhibited the Activation of NF-κB in Mouse Macrophages

**Figure 6:**
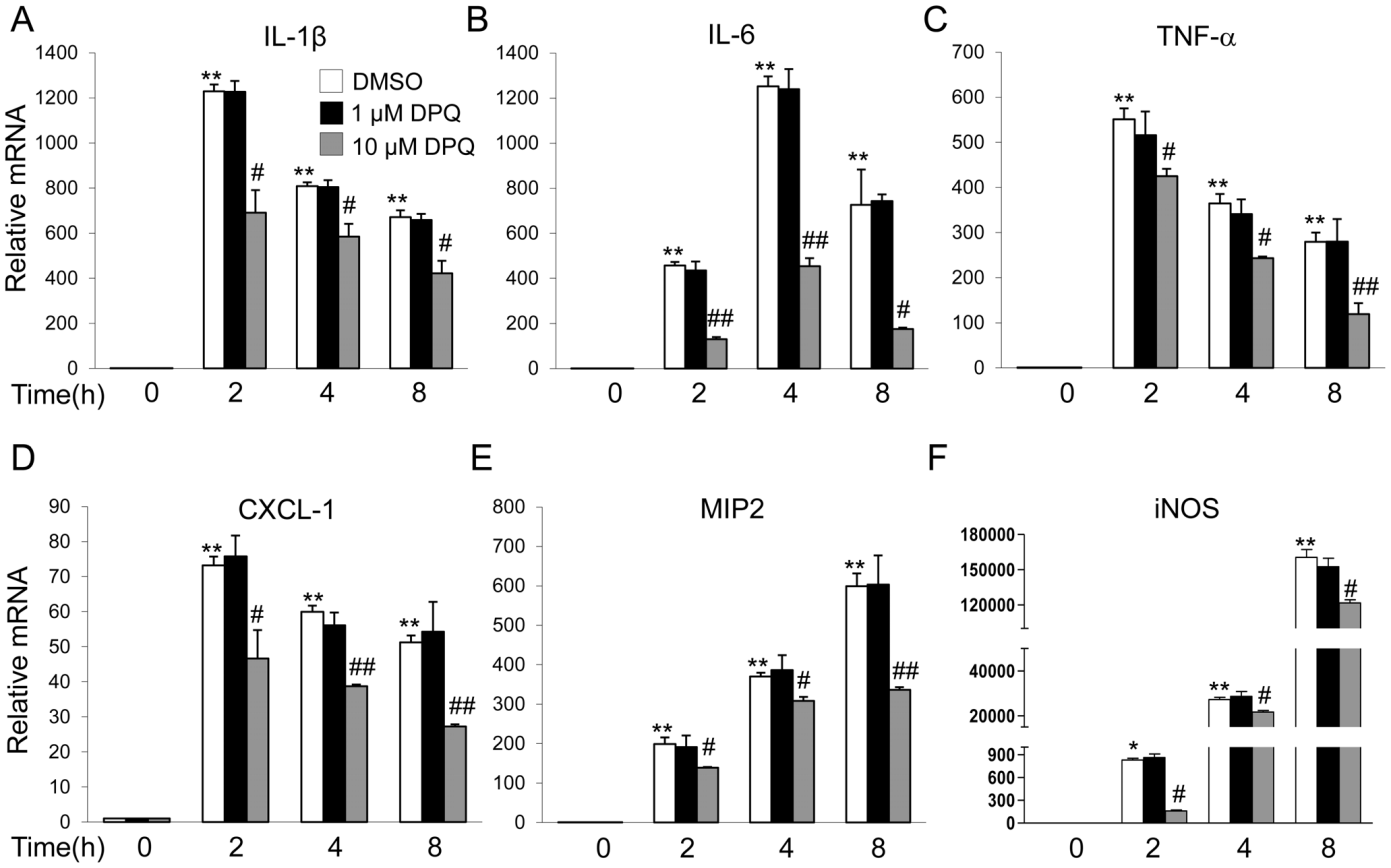
Real-time PCR analysis of the inflammatory mediators and cytokines mRNA expression in mouse macrophages. Macrophages isolated from mouse peritoneal cavity were cultured overnight and stimulated with 100/ml LPS for indicated time and lysed for RNA isolation. Real-time PCR analysis was performed to detect the mRNA expressions. Data are presented as mean ± SEM; n = 5; **P<0.01 versus Basal; #*P*<0.05 and ##*P*<0.01 versus LPS alone.

Macrophage has been demonstrated to play a pivotal role in lung inflammation, and depletion of functional macrophages in mouse leads to low lethality after challenged by lethal dose of LPS [27]. To further clarify the effects of DPQ on the macrophage-regulated inflammation, we isolated peritoneal macrophages and determined the cytokine expressions of macrophages challenged by 100 ng/ml of LPS. The expressions of pro-inflammatory mediators, including TNF-α, IL-1β, IL-6, MIP-2, iNOS and CXCL-1, were significantly induced, starting from 2 h post-LPS treatment. The pretreatment of DPQ at 10 μM partly blocked the up-regulations of these mediators, while DPQ at 1 μM did not show any inhibitory effects (**Fig. 6**).

**Figure 7:**
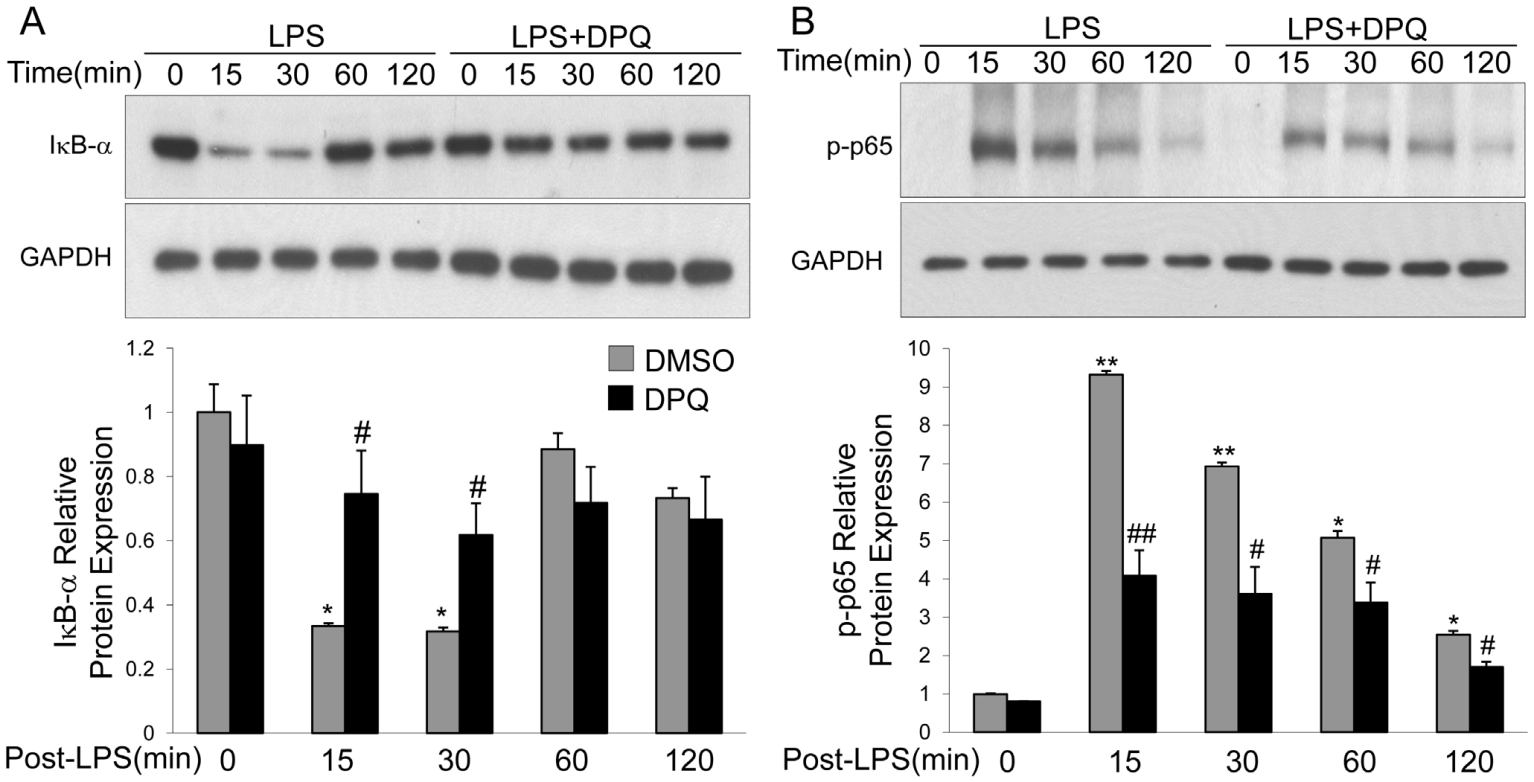
DPQ inhibits degradation of IκB-α and subsequent activation of NF-κB in macrophages. Macrophages were stimulated with LPS for indicated time and lysed for protein extraction. The expressions of IκB-α (A) and phosphorylation of NF-κB p65 (B) were detected by Western blot. The experiment was repeated for 3 times with similar results. Upper panel shows the representative immunoblot and lower panel shows the quantitative analysis of the protein expression. Data are expressed as mean ± SEM; n = 3 *P<0.05 and **P<0.01 versus Basal; #*P*<0.05 and ##*P*<0.01 versus LPS alone.

We also examined the activation of NF-κB signaling in the DPQ- or DMSO-pretreated macrophages following LPS challenge by western blot. The expression of IκB-α was decreased at 15 and 30 minutes and returned to basal level after 60 minutes following LPS treatment. Pretreatment of DPQ partially reversed the down-regulation of IκB-α at 15 and 30 minutes (**Fig. 7A**). Accordingly, the phosphorylation of NF-κB p65 subunit was significantly induced at 15, 30 and 60 minutes following LPS challenge, while DPQ partly blocked its up-regulation at indicated time points(**Fig. 7B**).

## Discussion

In the present study, we addressed that the potent and selective PARP-1 inhibitor, DPQ, protected mice against LPS-induced lung inflammation and lung injury. DPQ treatment inhibited the neutrophil infiltration and cell apoptosis in mice lungs following LPS challenge. In addition, DPQ restored the vascular integrity, as evidenced by EBA extravasation assay. LPS induced up-regulations of pro-inflammatory mediators and cytokines in mice and isolated peritoneal macrophages, which were partially inhibited by DPQ. Furthermore, DPQ treatment inhibited the degradation of IκB-α and subsequent activation of NF-κB in mice and macrophages. These results demonstrate that pharmacological inhibition of PARP-1 by DPQ exerts a protective effect on ALI via inhibiting NF-κB-mediated inflammatory response, consistent with the previous findings that inhibition or genetic deletion of PARP-1 protects the animals against endotoxin-induced lung injury or polymicrobial sepsis [28], [29], [30], [31], [32].

ALI in animals are characteristic of excessive neutrophil infilatration, release of pro-inflammatory, cytoxic mediators and loss of vascular barrier integrity [1]. Activated neutrophils induced extensive lung inflammation and contribute to the destruction of basement membrane and increased the permeability of alveolar-capillary membrane [10], [33]. Migrating neutrophils result in the mechanical damage to alveolar structure and worsen the influx of fluid into alveolar space [34]. In addition, neutrophils can release damaging mediators, such as oxidants and elastase, leading to the injury of epithelial-vascular barrier [35], [36], [37]. In this study, we found LPS induced significant neutrophil infiltration in lung interstitial space and enhanced neutrophil migration by MPO activity assay, consistent with the observations that neutrophil migration is the featuring hallmark in the development of ALI induced by LPS [10], herein DPQ treatment diminished neutrophil recruitment in lung tissue which may due to the decreased expression of neutrophil chemokines of CXCL-1 and MIP-2. Furthermore, we detected less EBA content and decreased cell apoptosis in mice treated with DPQ, showing the protective effect of DPQ against lung vascular injury. Taken together, our results demonstrated that DPQ exerted beneficial influence on lung inflammation and vascular injury after LPS challenge.

Except for neutrophils and the other inflammatory cells, the release of pro-inflammatory mediators has been reported to be involved in inflammatory cascade [1], [38]. Among these inflammatory mediators, cytokine ILs, such as TNF-α, IL-1β, IL-6, play critical roles in early phase of ALI. It has been reported that resident alveolar macrophages release TNF-α and IL-1β in early phase of inflammation in response to the pathogen stimulus, resulting in the subsequent inflammatory cascade and tissue injury [39], [40]. For example, TNF-α is reported to elevate intracellular reactive oxygen species which contributes to mitochondrial damage [41] or abnormal ion exchange across the cell membrane [42]. Inhibition of TNF-α by its mRNA transcription inhibitor or pharmacological inhibitor exhibits protective effects in preclinical models of ALI [43], [44]. IL-1β can induce lung fibrosis and release of a variety of chemokines, including monocyte chemotatic protein-1, MIP-1α [45]. IL-1β also enhances the recruitment of inflammatory cells into airspaces and alters vascular permeability, leading to fluid transport and subsequent lung edema formation [46], [47]. Elevated TNF-α and IL-1β in plasma is predictive of clinical outcome [1]. In the present study, the expressions of TNF-α, IL-1β, IL-6, and iNOS were markedly induced by LPS challenge, which was blocked by the treatment of DPQ. Our results confirmed the link between the inflammatory cytokine levels and the extent of lung inflammation, demonstrating the anti-inflammatory effects of DPQ *in vivo*.

On the other hand, since macrophages mediate the systemic effects of LPS, we isolated the peritoneal macrophages to investigate the effects of DPQ *in vitro*. Upon stimulation by LPS, macrophages increase the production of TNF-α, IL-1β and IL-6 via activating hypoxia inducible factor-1α (HIF-1α), while knockdown of HIF-1α suppresses the production of these cytokines [48], [49], suggesting the correlation of cytokines production and LPS challenge in macrophages. In this study, we found DPQ at 10 μM partly inhibited the expressions of pro-inflammatory mediators and cytokines in macrophages following LPS challenge, suggesting the anti-inflammatory effect of DPQ *in vitro*. Taken together, these data confirmed the involvement of macrophages in the mediation of lung inflammation, suggesting DPQ attenuates ALI by inhibiting the activation of macrophages and production of inflammatory mediators.

In tissue inflammatory and immune response, NF-κB signaling plays a central role via transcriptionally regulating gene expressions [50]. In experimental animal models of ALI, NF-κB activation is increased [49], [51]. Pharmacological inhibition of NF-κB pathway shows decreased production of pro-inflammatory mediators and protective effects against endotoxin-induced ALI in animals [51], [52]. In the present study, we found LPS challenge induced the degradation of IκB-α and activation of NF-κB *in vivo* and *in vitro*, and the activation of NF-κB signaling was inhibited by DPQ treatment, suggesting that inhibition of NF-κB signaling plays a role in the protective effects of DPQ on ALI. It has been reported that NF-κB signaling can be activated by several intracellular signaling pathways which are regulated by PARP-1 [53], [54], [55]. For example, following inflammatory stimuli, PARP-1 is activated by the damaged DNA induced by oxidative stress [56], [57], and then blocked the interaction of NF-κB and Crm-1 which regulates p65 NF-κB nuclear translocation [58], [59], leading to the failure of NF-κB activation. Intriguingly, we found DPQ did not fully inhibited the activation of NF-κB, which can be explained by the findings that NF-κB can also be activated by the other signals, such as oxidative stress [58], [60]. Thus, our results are consistent with the inhibitory effects of NF-κB activation by PARP-1 inhibitor and confirm that DPQ blocks the activation of NF-κB by inhibiting PARP-1 activity.

In conclusion, we have demonstrated the protective effects of DPQ on acute lung injury induced by LPS, as evidenced by alleviative lung inflammation, decrease of vascular leakage and pro-inflammatory cytokine release, inhibition of NF-κB activation and cell apoptosis. Our study indicates that pharmacological inhibition of PARP-1 might be a potential approach in the therapy of ALI.
